# Changes of Aboveground and Belowground Biomass Allocation in Four Dominant Grassland Species Across a Precipitation Gradient

**DOI:** 10.3389/fpls.2021.650802

**Published:** 2021-04-13

**Authors:** Yongjie Liu, Mingjie Xu, Guoe Li, Mingxia Wang, Zhenqing Li, Hans J. De Boeck

**Affiliations:** ^1^State Key Laboratory of Grassland Agro-Ecosystems, Key Laboratory of Grassland Livestock Industry Innovation, Ministry of Agriculture and Rural Affairs, College of Pastoral Agriculture Science and Technology, Lanzhou University, Lanzhou, China; ^2^State Key Laboratory of Vegetation and Environmental Change, Institute of Botany, Chinese Academy of Sciences, Beijing, China; ^3^University of Chinese Academy of Sciences, Beijing, China; ^4^Plants and Ecosystems (PLECO), Department of Biology, University of Antwerp, Wilrijk, Belgium

**Keywords:** aboveground biomass, belowground biomass, climate change, grassland, grassland persistence, rangeland sustainability, precipitation amount

## Abstract

Climate change is predicted to affect plant growth, but also the allocation of biomass to aboveground and belowground plant parts. To date, studies have mostly focused on aboveground biomass, while belowground biomass and allocation patterns have received less attention. We investigated changes in biomass allocation along a controlled gradient of precipitation in an experiment with four plant species (*Leymus chinensis*, *Stipa grandis*, *Artemisia frigida*, and *Potentilla acaulis*) dominant in Inner Mongolia steppe. Results showed that aboveground biomass, belowground biomass and total biomass all increased with increasing growing season precipitation, as expected in this water-limited ecosystem. Biomass allocation patterns also changed along the precipitation gradient, but significant variation between species was apparent. Specifically, the belowground biomass: aboveground biomass ratio (i.e., B:A ratio) of *S. grandis* was not impacted by precipitation amount, while B:A ratios of the other three species changed in different ways along the gradient. Some of these differences in allocation strategies may be related to morphological differences, specifically, the presence of rhizomes or stolons, though no consistent patterns emerged. Isometric partitioning, i.e., constant allocation of biomass aboveground and belowground, seemed to occur for one species (*S. grandis*), but not for the three rhizome or stolon-forming ones. Indeed, for these species, the slope of the allometric regression between log-transformed belowground biomass and log-transformed aboveground biomass significantly differed from 1.0 and B:A ratios changed along the precipitation gradient. As changes in biomass allocation can affect ecosystem functioning and services, our results can be used as a basis for further studies into allocation patterns, especially in a context of environmental change.

## Introduction

Climate change is affecting rainfall patterns in many regions around the world (Arnbjerg-Nielsen et al., 2013; Ohba and Sugimoto, 2019; Hyun and Yeh, 2020). Such changes can significantly alter plant growth and vegetation dynamics, both when precipitation amounts decrease or when they increase (Felton et al., 2019). Drought triggers mostly neutral or negative responses regarding growth and biomass (Zhang et al., 2012; Gherardi and Sala, 2019; Meng et al., 2019), while increased precipitation mostly leads to neutral or positive growth responses (Chu et al., 2016; Michaletz et al., 2018; Gherardi and Sala, 2019). While these are general patterns, where the biomass ends up under any response scenario (decreased, increased, or unchanged biomass) is also relevant as this can affect, for example, livestock feeding, soil stability, and carbon sequestration (Herrero et al., 2013; Maryol and Lin, 2015; Reinhart and Vermeire, 2017).

The optimal partitioning theory predicts that plants tend to allocate relatively more biomass to organs increasing the uptake of the most limiting resources (Bloom et al., 1985; Gedroc et al., 1996; Mao et al., 2012). Therefore, plants are expected to allocate more biomass belowground under dry conditions, and more aboveground when growing under wet conditions (Villar et al., 1998). The isometric partitioning theory suggests that aboveground biomass and belowground biomass follows an isometric pattern (Enquist and Niklas, 2002; Wang et al., 2014), implying that there is not necessarily a trade-off between aboveground and belowground. However, contrasting results have been found, with both studies in support (e.g., Enquist and Niklas, 2002; Wang et al., 2014) and studies that rejected isometric partitioning (e.g., Chen et al., 2016; Ma and Wang, 2021). Thus, further studies are needed to shed more light on this theory.

While it is clear that environmental changes can significantly affect biomass allocation (Fan et al., 2009; Zhang et al., 2017; Yang et al., 2018; Zhou et al., 2020), most studies that explored the effects of climate change on biomass allocation have focused on aboveground biomass (Bai and Xu, 1997; Mokany et al., 2006; Bai et al., 2008; Gonzalez-Dugo et al., 2010). Few studies include belowground biomass as this is more difficult to measure, especially in the field (Milchunas et al., 2005; Ma et al., 2008). Therefore, our knowledge of changes in plant allocation pattern triggered by changes in the environment is generally incomplete (Achten et al., 2010; Liu et al., 2015) and exact allocation strategies merit further investigation (Pan et al., 2005; Cai et al., 2005; Lv et al., 2016).

Grasslands, as one of the main terrestrial ecosystems, occupy more than 30% of the terrestrial area (Parton et al., 2012). They play an important role in biogeochemical cycles and energy transformation (Huang et al., 2010; Bai et al., 2012). Compared with forests, grasslands show more pronounced responses to climate change, at least in the short term (Eziz et al., 2017; Maurer et al., 2020), and are thus a relevant ecosystem to study in the context of environmental change. In grasslands, biomass allocation is a key mechanism for understanding the dynamics involved in plant growth, and changes therein can alter the structure and functioning of these systems (Poorter et al., 2012a,b).

To improve the knowledge on changes in biomass allocation patterns under varying environmental conditions in grasslands, we conducted an experiment to explore effects of growing season precipitation on biomass aboveground and belowground. We focused on four plant species (i.e., *Leymus chinensis*, *Stipa grandis*, *Artemisia frigida*, and *Potentilla acaulis*) dominant in Inner Mongolia steppe, and applied a gradient including eight levels of precipitation centered around the local annual mean precipitation. *L. chinensis* is a perennial forage grass with long strong rhizomes, *S. grandis* is a perennial tussock grass with closely clumped shoots, while *A. frigida* and *P. acaulis* are perennial herbs with stolons and developed adventitious roots (Li et al., 2005; Liu et al., 2006, 2007). The objective of this study was to test the optimal partitioning theory and the isometric partitioning theory at the species scale. Specially, we aimed to explore the relationships between precipitation amount and aboveground biomass, belowground biomass, total biomass and belowground biomass: aboveground biomass (B:A) ratio. Previous studies found that species with rhizomes or stolons tended to allocate more biomass to roots (i.e., belowground) (Schmid, 1987; Enquist and Niklas, 2002; Rhazi et al., 2009), leading to hypothesis (1), namely that the B:A ratio of *L. chinensis*, *A. frigida*, and *P. acaulis* is expected to be larger than that of *S. grandis*. Furthermore, if species with rhizomes or stolons indeed allocate more biomass belowground, they may respond differently along a gradient of changing precipitation compared to other species, according to the optimal partitioning theory. Under this hypothesis (2) the B:A ratios of *L. chinensis*, *A. frigida*, and *P. acaulis* would increase with precipitation amount, while a different pattern may be apparent in *S. grandis*. However, under the isometric partitioning hypothesis (3), the B:A ratios of these species are expected to be constant with precipitation amount (Enquist and Niklas, 2002; Yang and Luo, 2011; Wang et al., 2014). This same hypothesis also states that aboveground biomass should be scale with belowground biomass across our dataset.

## Materials and Methods

### Field Site

This study was conducted on Inner Mongolia steppe in China (43°33′N, 116°40′E), where the mean elevation ranges from 1,200 to 1,250 m. Local climate is characterized by a mild humid summer and a dry cold winter, with the mean annual temperature (MAT) ranging from −1.1 to 0.2°C, and large seasonal differences (−21.4°C on average in the coldest month, January, and 18.5°C on average in the warmest month, July). Mean annual precipitation (MAP) is 350 mm (from 1980 to 2000), of which around 280 mm falls in the growing season.

### Experimental Design

To explore biomass allocation to aboveground and belowground plant parts, a manipulation experiment was conducted from May 2000 to October 2001. Four plant species dominant in the Inner Mongolia steppe were subjected to eight levels of growing season precipitation (administered through watering), centered around the local MAP (i.e., 350 mm): 170, 250, 300, 350, 525, 595, 665, and 700 mm. Such a large gradient enabled us to explore the effects of precipitation (including both dry and wet conditions) on plant biomass and biomass allocation, and was not intended to mimic the variation of local rainfall expected under climate change (cf. Kayler et al., 2015). Our experiment was conducted in a plot with a rainout shelter in order to block natural rainfall. This shelter was covered by highly transparent plastic foil upward from 2 m above the ground in order to prevent warming and to allow wind circulation. The impact on temperature, air humidity and light with such a design is limited (Kreyling et al., 2017).

Plants were grown in pots of 50 cm height and 30 cm diameter, filled with soil collected from nearby grasslands (mainly dark chestnut soil with a thin humus layer, cf. Li and Li, 2002; Jia et al., 2005). We used soil from the top 50 cm, which was well mixed and from which roots were carefully removed. There were three replications of each treatment for each species. For *L. chinensis*, seeds were randomly sown in the pots in early May 2000, and four similar-sized individuals were retained after germination. For *S. grandis*, four ramets with similar size were transplanted into each pot in late May 2000 following unsuccessful seed germination in early May. For *A. frigida* and *P. acaulis*, plants were excavated and ramets were separated into similar size. Four of them were transplanted into each pot in early May 2001. All the plants were first grown in an open air area under natural conditions, and rainout shelters were deployed and treatments were applied from 10 June to 10 September 2001. During the experiment, water was added daily to each pot, with the water amount determined by dividing the total amount of precipitation amount in each treatment by the total growing days. To reduce water runoff, water was evenly added by hand at the soil surface. Note that around 80% of the annual rainfall occurs from June through August. The watering we provided thus covered most of the annual precipitation in line with previous studies (Hagiwara et al., 2010).

At the end of the experiment, all plants were washed free of soil with distilled water, and separated into aboveground and belowground parts. For *L. chinensis* and *S. grandis*, aboveground parts included leaves and stems, while belowground parts included roots and rhizomes. For *A. frigida*, aboveground parts included leaves, flowers, and stems, while belowground parts included roots. Finally, for *P. acaulis*, aboveground parts included leaves and stems, while belowground parts included roots. All of these were oven-dried at 65°C to constant weight and subsequently weighed.

### Statistical Analysis

Aboveground and belowground biomass per square meter was calculated by dividing biomass of the four individuals in each pot by the surface area of each pot. Total biomass relates to the sum of aboveground and belowground biomass and the belowground biomass: aboveground biomass ratio (i.e., B:A ratio) was calculated by dividing belowground biomass by aboveground biomass.

Two-way analysis of variance (ANOVA) was conducted to explore the effects of species, precipitation amount and their interaction on the aboveground biomass, belowground biomass, total biomass and B:A ratio. *Post hoc* analysis (pairwise comparisons with Bonferroni corrections) was applied to test the differences among the target plant species. One data point of aboveground biomass of *S. grandis* at 700 mm precipitation was identified as an outlier and was removed. All statistics were carried out using SPSS 21.0.

Curve estimations were done to test the relationships between precipitation amount and aboveground biomass, belowground biomass, total biomass and B:A ratio, where linear, quadratic, power and exponential curves were tested. AIC (Akaike Information Criterion) and *P* value were used to identify better models, i.e., lower AIC and significant (and lower) *P* value (Cottingham et al., 2005).

The relationship between log-transformed belowground biomass and log-transformed aboveground biomass across the precipitation gradient was determined with ordinary least square regression and standardized major axis regression (Niklas, 2005; Cheng and Niklas, 2007). The slopes were tested against the 1:1 line, where non-significant difference indicates an isometric relationship between belowground and aboveground biomass. Slopes and intercepts were obtained with a software package developed by Falster et al. (2006).

## Results

**FIGURE 1:**
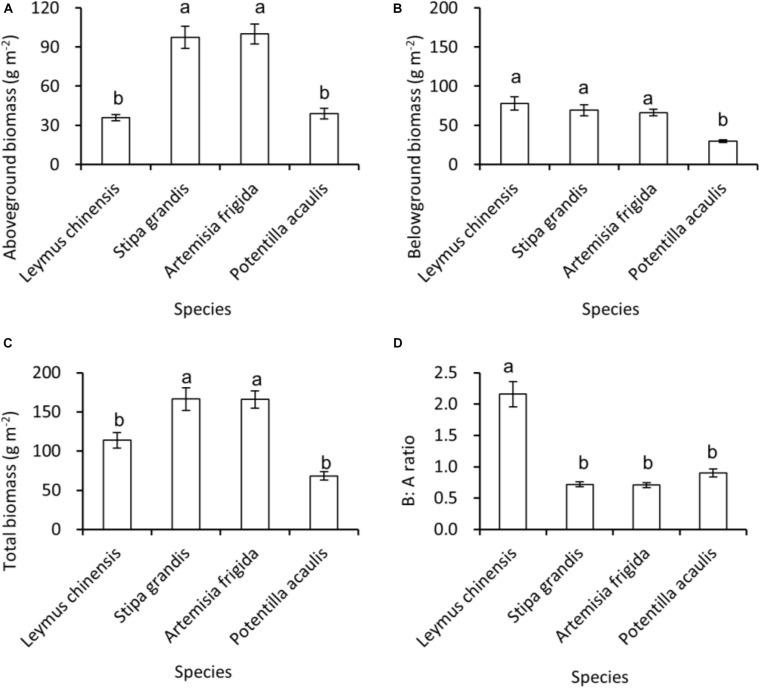
At the pot scale, mean ± SE of **(A)** aboveground biomass, **(B)** belowground biomass, **(C)** total biomass, and **(D)** B:A ratio (i.e., ratio of belowground biomass and aboveground biomass), across the precipitation gradient, per species, i.e., *Leymus chinensis*, *Stipa grandis*, *Artemisia frigida*, and *Potentilla acaulis*. Significant (*P* < 0.05) differences between species have different letters (*post hoc* analyses with Bonferroni corrections).

**FIGURE 2:**
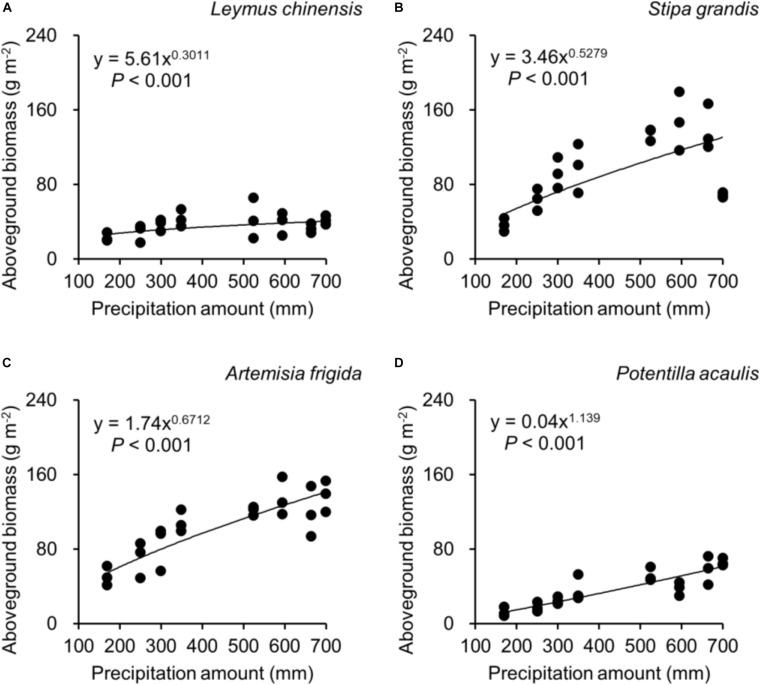
At the pot scale, regressions between precipitation amount and aboveground biomass, separately for **(A)** *Leymus chinensis*, **(B)** *Stipa grandis*, **(C)** *Artemisia frigida*, and **(D)** *Potentilla acaulis.*

**FIGURE 3:**
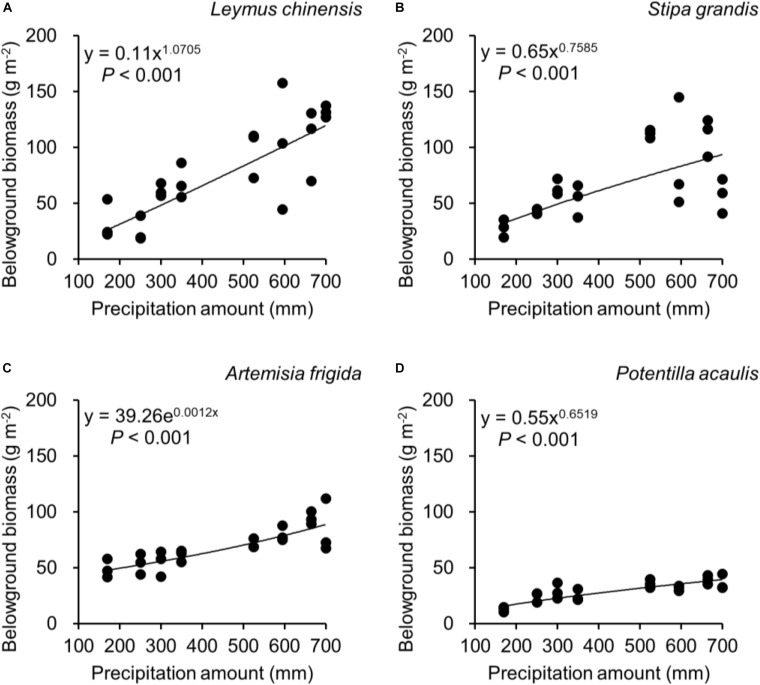
At the pot scale, regressions between precipitation amount and belowground biomass, separately for **(A)** *Leymus chinensis*, **(B)** *Stipa grandis*, **(C)** *Artemisia frigida*, and **(D)** *Potentilla acaulis.*

**FIGURE 4:**
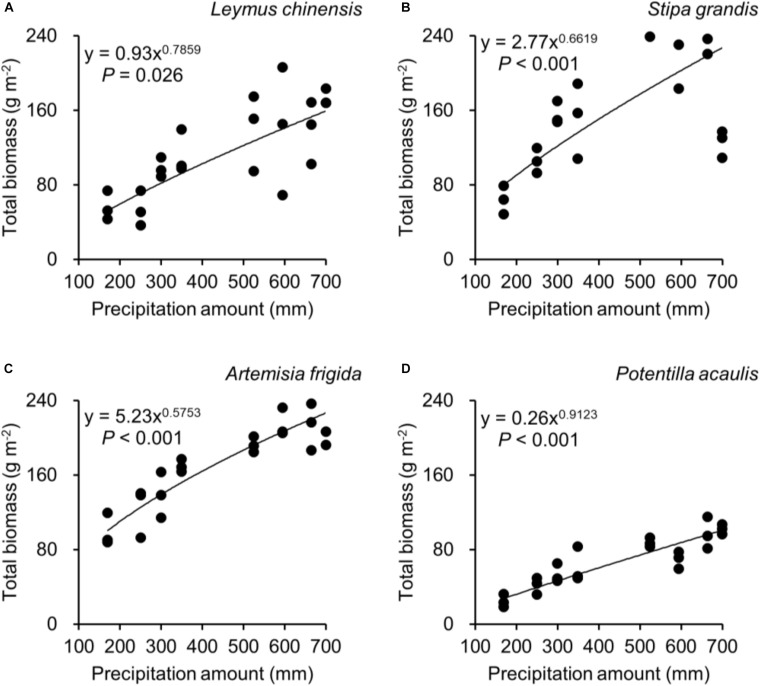
At the pot scale, regressions between precipitation amount and total biomass, separately for **(A)** *Leymus chinensis*, **(B)** *Stipa grandis*, **(C)** *Artemisia frigida*, and **(D)** *Potentilla acaulis*.

**FIGURE 5:**
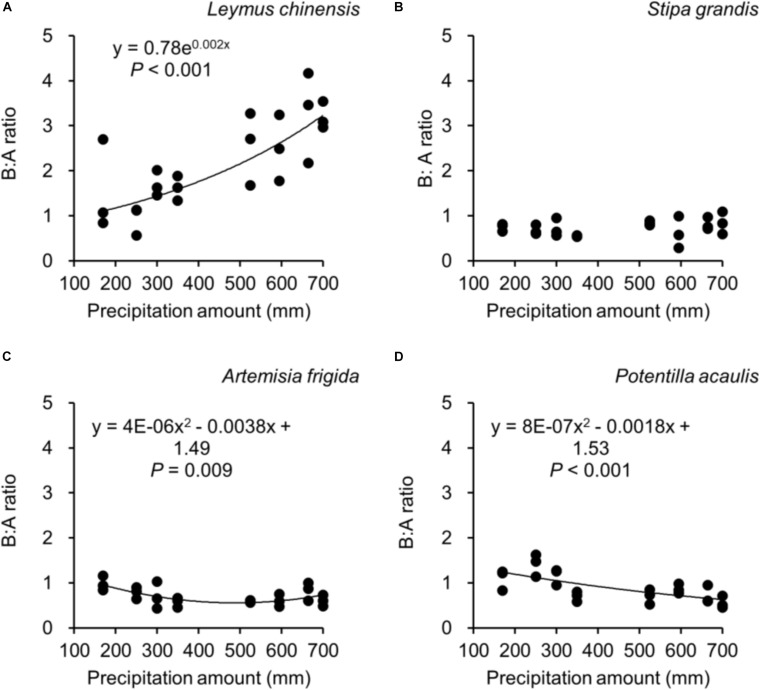
At the pot scale, regressions between precipitation amount and B:A ratio (i.e., ratio of belowground biomass and aboveground biomass), separately for **(A)** *Leymus chinensis*, **(B)** *Stipa grandis*, **(C)** *Artemisia frigida*, and **(D)** *Potentilla acaulis*, where each dot refers to a B:A ratio from a pot.

Regarding species differences, *S. grandis* and *A. frigida* on average had more aboveground (Figure 1A) and total biomass (Figure 1C) than *L. chinensis* and *P. acaulis*. Meanwhile, *P. acaulis* had a lower belowground biomass than the other three plant species (Figure 1B). Interestingly, *L. chinensis* had a larger B:A ratio than the other species (Figure 1D). Precipitation amount significantly affected aboveground biomass, belowground biomass, total biomass and B:A ratio and these effects differed amount the target plant species (Table 1). Moreover, significant interactive effects of species and precipitation amount on the aboveground biomass, belowground biomass, total biomass and B:A ratio were found (Figures 2–5 and Table 2). Specially, positive patterns were found in relationships between precipitation amount and (i) aboveground biomass (Figure 2), (ii) belowground biomass (Figure 3), and (iii) total biomass (Figure 4).

**TABLE 1:** Effects of species, precipitation amount and their interaction in two-way ANOVA on aboveground biomass, belowground biomass, total biomass, and B:A ratio (i.e., ratio of belowground biomass and aboveground biomass).

	**Aboveground biomass**			**Belowground biomass**		
	**df**	***F***	***P***	**df**	***F***	***P***
SpeciesPrecipitation amountSpecies × Precipitation amount	3.647.6421.64	104.59523.2522.665	**<0.001<0.0010.001**	3.647.6421.64	36.57117.4122.817	**<0.001<0.0010.001**
	
	**Total biomass**			**B:A ratio**		
	**df**	**F**	***P***	**df**	**F**	***P***
	
SpeciesPrecipitation amountSpecies × Precipitation amount	3.647.6421.64	89.81933.4793.606	**<0.001<0.001<0.001**	3.647.6421.64	33.6232.1393.023	**<0.001**0.052**<0.001**

*Significant differences are indicated in bold.*

**TABLE 2:** Results of the curve estimation of the relationships between precipitation amount and aboveground biomass, belowground biomass, total biomass, and B:A ratio (i.e., ratio of belowground biomass and aboveground biomass) of *Leymus chinensis*, *Stipa grandis*, *Artemisia frigida*, and *Potentilla acaulis* with linear, quadratic, power, and exponential equations, where AIC, F, df, and *P* value were showed.

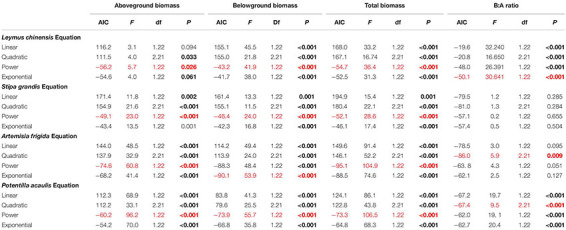

*A better estimation (marked in red) is determined by a smaller AIC (Akaike Information Criterion) and a significant *P* value (marked in bold).*

Along the precipitation gradient, we observed different B:A ratios in the four target plant species. Increasing precipitation did not significantly affect the B:A ratio of *S. grandis* (Figure 5B), while it increased the B:A ratio of *L. chinensis* (Figure 5A), decreased for *P. acaulis*, and seemingly first decreased and then increased for *A. frigida* (with a threshold around 475 mm). A greater B:A ratio suggests a greater biomass investment in the belowground organs.

**FIGURE 6:**
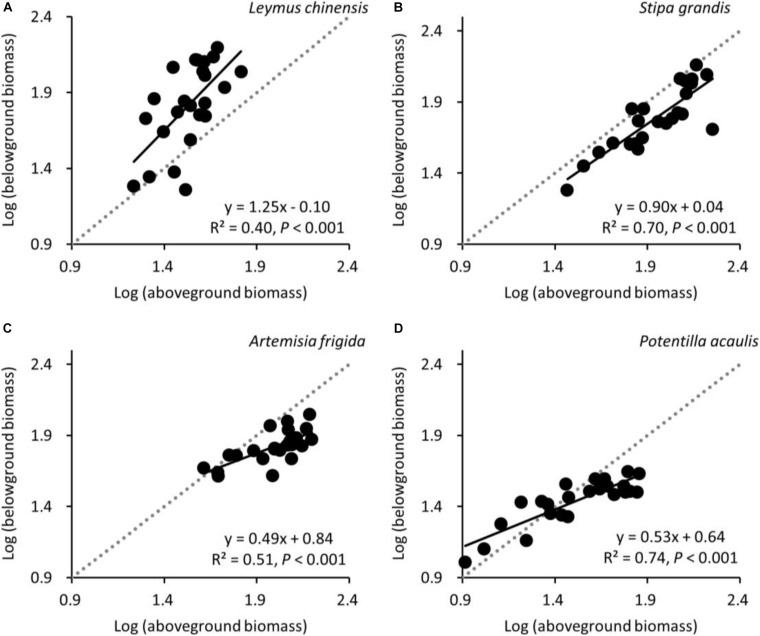
At the pot scale, allometric relationships between log-transformed aboveground biomass and long-transformed belowground biomass for **(A)** *Leymus chinensis*, **(B)** *Stipa grandis*, **(C)** *Artemisia frigida*, and **(D)** *Potentilla acaulis*. The 1:1 line (dotted) is added for clarity.

Aboveground biomass was positively correlated with belowground biomass for all four target species, as expected (Figure 6). The slopes of the relationship between log-aboveground biomass and log-belowground biomass for *L. chinensis*, *S. grandis*, *A. frigida*, and *P. acaulis* were 1.25, 0.90, 0.49, and 0.53, respectively. These values differed significantly from 1.0 for three species (*P* = 0.001, <0.001, and <0.001 for *L. chinensis*, *A. frigida*, and *P. acaulis*, respectively), indicating non-isometric growth for these rhizome or stolon-forming species. The relationship did not differ significantly from the 1:1 line for *S. grandis* (*P* = 0.275).

## Discussion

In this study, we subjected four species common in grasslands of Inner Mongolia to a precipitation gradient. In general, we found that both aboveground biomass and belowground biomass production was stimulated as growing season precipitation was increased. This was unsurprising, given that these grasslands are known to be precipitation-limited (Kang et al., 2011; Guo et al., 2015). The focus in the current study was primarily on biomass allocation patterns, which we considered by testing three hypotheses. The first hypothesis stated that species with rhizomes or stolons would allocate more biomass belowground. This pattern was only found for one rhizome and stolon forming species, namely *L. chinensis*. The other two such species, *A. frigida* and *P. acaulis*, displayed similar allocation patterns with the non-rhizome or stolon forming *S. grandis*. It should be noted that in contrast to studies calculating biomass allocation based on root biomass (e.g., Berendse and Möller, 2009), we considered the complete belowground biomass, including roots, rhizomes, and stolons.

The second hypothesis studied here, assumed that biomass allocation of species with rhizomes or stolons would increase along the precipitation gradient. This was not convincingly supported, with different patterns between precipitation amount and B:A ratios being observed for the four target plant species. Specifically, the B:A ratio of non-rhizome or stolon forming *S. grandis* remained constant along the precipitation gradient, suggesting that the biomass allocation of this species was not sensitive to precipitation amount. In line with our expectations, a positive pattern was found in *L. chinensis*, which could be explained by the fact that *L. chinensis* has a strong forage ability as a rhizomatous species (Wang et al., 2004), which enables it to allocate more biomass to roots when growing in wet conditions (Yang and Yang, 1998). Similar patterns were also found in species such as *Salix psammophila*, *Hedysarum leave*, *Artemisia ordosica*, and *Caragana korshinskii* (Dong et al., 1999; Xiao et al., 2001). Nevertheless, a contrasting (negative) pattern was apparent for *P. acaulis*, indicating more biomass was allocated aboveground with increasing precipitation amount. Interestingly, our data suggested a unimodal pattern between precipitation amount and B:A ratio for *A. frigida*, with higher precipitation only increasing the biomass allocation belowground up to a certain point.

According to isometric partitioning, aboveground biomass and belowground biomass would be isometric at the species scale (hypothesis 3). This would suggest both no changes in B:A ratios along the precipitation gradient and no deviation from 1:1 lines in the aboveground biomass vs. belowground biomass relationship. Our results suggest that only *S. grandis* seemed to respond in line with isometric partitioning. The three rhizome or stolon-forming species did not adhere to isometric partitioning, with both asymmetrical variation between aboveground biomass and belowground biomass, in contrast with Enquist and Niklas (2002) and Yang et al. (2009), as well as differences in B:A ratios along the precipitation gradient. Regarding the allometric relationships between aboveground biomass and belowground biomass, the average slope of the four target plant species was 0.79, which is in line with the global grasslands’ slope (i.e., 0.72, Wang et al., 2014), but smaller than China’s grasslands’ slope (i.e., 1.05, Wang et al., 2014). Such differences may be caused by the limited number of plant species used in this study, and because we explored allometric partitioning at the species scale, not at the individual or the community scale like in previous studies (Enquist and Niklas, 2002; Wang et al., 2010, 2014).

Biomass allocation between belowground biomass and aboveground biomass differed among species in our study, in line with previous findings (Ma et al., 2008; Kang et al., 2013; Gong et al., 2015; Zhang et al., 2019). Mokany et al. (2006) suggested that the root/shoot (R/S) ratio in grasslands tends to decrease with increasing MAP. However, Yang et al. (2010) reported that the R/S ratio in China’s grasslands did not show any significant pattern along increasing MAP. Several potential causes were proposed, relating to climatic factors (e.g., MAT and MAP). The plant species used in our experiment, which are dominant species in the Inner Mongolia steppe, displayed various relationships between B:A (similar to R/S) ratio and precipitation amount. Plant communities with species responding differently regarding biomass allocation, e.g., in an opposite direction, to precipitation may see little total effect at the community scale. Nevertheless, the species-specific changes in allocation patterns could lead to different competitive outcomes (Aerts et al., 1991), thus changing species composition in the longer term, and thus the B:A (or R: S) ratio of the community.

Results of this study should be interpreted and extrapolated with caution for a number of reasons. First, the experiment was short term, featuring a limited number of species. Studying longer term effects on more species would allow more extensive generalization. Furthermore, two species in this study were sown at the beginning of the experiment, while the other two were transplanted from local grasslands. It is possible that plant age affects allocation patterns, with for example Yu et al. (2019) reporting that resource limitation could be partially the reason of decreasing allocation with age, where resources such as nutrients and waters become limited with plant grow (age). Moreover, we allowed intraspecific competition in our study, which is realistic, but which would also alter allocation patterns (Yang et al., 2019). Comparisons with experiments considering individual plants (e.g., Lamb et al., 2007), are thus not straightforward. Another factor to consider in future studies is soil heterogeneity. Plants may allocate more biomass to roots when growing in higher levels of soil heterogeneity (James et al., 2003; Michael and Elizabeth, 2004; Hagiwara et al., 2010; Wu et al., 2014; Liu et al., 2017a), and plants growing on low-nutrient patches have been reported to grow more roots into their neighboring high-nutrient patches (Liu et al., 2017b, 2019).

In sum, in our experiment we found that changes in precipitation affected biomass allocation in general, but that significant species-specific differences were apparent. Increasing precipitation increased the biomass allocation to belowground organs for one species with rhizomes or stolons, while it did not impact the biomass allocation of the non-rhizomes or stolon-forming species in our study. Isometric partitioning, meaning constant allocation of biomass aboveground and belowground regardless of plant size or precipitation amounts, seemed to occur for one species, but not for the rhizome or stolon-forming ones. Increased knowledge of allocation patterns leads to improved understanding of the structure and functioning of grasslands under changes in the environment, such as altered precipitation. Moreover, changed allocation patterns matter as they can affect agricultural value, carbon sequestration, and climate resilience. The results of our study could be used as a basis for further research into allocation patterns in a changing environment, spanning a wider range of species, and explicitly considering consequences for ecosystem services.

## Data Availability Statement

The original contributions presented in the study are included in the article/supplementary material, further inquiries can be directed to the corresponding author.

## Author Contributions

ZL designed and conducted the study. YL and ZL analyzed the data. All authors discussed the data and contributed crucially to the drafts.

## Conflict of Interest

The authors declare that the research was conducted in the absence of any commercial or financial relationships that could be construed as a potential conflict of interest.
